# Expression of indoleamine 2.3-dioxygenase by tumors induces local and systemic immunosuppressive effects in a murine melanoma model

**DOI:** 10.1186/2051-1426-2-S3-P212

**Published:** 2014-11-06

**Authors:** Rikke B Holmgaard, Dmitriy Zamarin, David H Munn, Taha Merghoub, Jedd D Wolchok

**Affiliations:** 1 Memorial Sloan Kettering Cancer Center, New York, NY, USA; 2 Cancer Center and Department of Pediatrics, Georgia Regents University, Augusta, GA, USA

## 

Indoleamine 2,3-dioxygenase (IDO) is found in the majority of human tumors and has been described as an important contributor to the development of an immunosuppressive tumor microenvironment that blocks the action of cytotoxic antitumor effector T cells. In order to delineate the mechanisms of IDO-induced immunosuppression in melanoma, we initially focused on murine transplantable and spontaneous melanoma models. We find that while murine melanomas did not naturally express high levels of IDO, IDO expression could be induced in the tumor cells within the context of the tumor microenvironment *in vivo*. In order to examine the role of IDO on the melanoma tumor microenvironment and its effect on tumor progression, we generated B16 melanoma cell lines overexpressing IDO (B16-IDO). When implanted into mice, B16-IDO tumors exhibited aggressive tumor growth, characterized by more rapid tumor progression and local invasion, when compared to the native B16 cells. Furthermore, constitutive expression of IDO by B16 cells conferred resistance to T-cell-targeting immunotherapies, such as treatment with CTLA-4 and PD-1 blocking antibodies. Interestingly, the immunosuppressive effect of IDO overexpression was evident not just within the tumor, but systemically as well. Analysis of the tumors *in vivo*, showed that this effect was accompanied by changes favoring a highly inhibitory tumor microenvironment characterized by significant reduction on local tryptophan concentrations and lack of effector T cells at the tumor site. The effect of IDO overexpression on immunosuppressive cell populations of the tumor microenvironment such as regulatory T cells and myeloid-derived suppressor cells will be further characterized. Our study thus defines IDO expression as an important mechanism driving the institution of an inhibitory tumor microenvironment and tumor progression, and provides a strong rationale for therapeutic targeting of this pathway.

